# European Society of Pediatric Radiology survey of perioperative imaging in pediatric liver transplantation: (3) postoperative imaging

**DOI:** 10.1007/s00247-023-05842-z

**Published:** 2024-01-29

**Authors:** Elena Dammann, Lil-Sofie Ording-Müller, Stéphanie Franchi-Abella, Martijn V. Verhagen, Simon P. McGuirk, Reinoud P.H. Bokkers, Philippe R. M. Clapuyt, Annamaria Deganello, Francesco Tandoi, Jean de Ville de Goyet, Hanna Hebelka, Charlotte de Lange, Cecile Lozach, Paolo Marra, Darius Mirza, Piotr Kaliciński, Janina M. Patsch, Giulia Perucca, Ilias Tsiflikas, Diane M. Renz, Bernd Schweiger, Marco Spada, Seema Toso, Loïc Viremouneix, Helen Woodley, Lutz Fischer, Florian Brinkert, Philippe Petit, Jochen Herrmann

**Affiliations:** 1 Section of Pediatric Radiology, Department of Diagnostic and Interventional Radiology and Nuclear Medicine, Universitätsklinikum Hamburg-Eppendorf, Martinistrasse 52, 20246 Hamburg, Germany; 2 Department of Pediatric Radiology, Rikshospitalet University Hospital: Oslo universitetssykehus Rikshospitalet, Oslo, Norway; 3 Department of Pediatric Radiology, Hôpital Bicêtre, Le Kremlin-Bicêtre, France; 4 Department of Radiology, University Medical Centre Groningen: Universitair Medisch Centrum Groningen, Groningen, Netherlands; 5 Department of Radiology, Birmingham Children’s Hospital, Birmingham, UK; 6 Department of Radiology, Cliniques Universitaires Saint-Luc, Brussels, Belgium; 7 Department of Radiology, King’s College Hospital, London, UK; 8 Department of Hepatobiliary and Transplant Surgery, Azienda Ospedaliero-Universitaria Città della Salute e della Scienza di Torino, Turin, Italy; 9 Department of Pediatrics and Pediatric Transplantation, ISMETT-UPMC, Palermo, Italy; 10 Department of Radiology, The Institute of Clinical Sciences, Gothenburg, Sweden; 11 Department of Pediatric Radiology, Queen Silvia Children’s Hospital, Gothenburg, Sweden; 12 Department of Radiology, Hôpital Universitaire Necker-Enfants-Malades, Paris, France; 13 Department of Radiology, Azienda Ospedaliera Ospedali Riuniti di Bergamo: Aziende Socio Sanitarie Territoriale Papa Giovanni XXIII, Bergamo, Italy; 14 Department of Hepatobiliary and Transplant Surgery, Birmingham Children’s Hospital, Birmingham, UK; 15 Department of Pediatric Surgery and Organ Transplantation, The Children’s Memorial Health Institute, Warsaw, Poland; 16 Department of Radiology, Medical University of Vienna, Vienna, Austria; 17 Department of Radiology, Great Ormond Street Hospital for Children, London, UK; 18 Department of Pediatric Radiology, Regina Margherita Children’s Hospital, Turin, Italy; 19 Department of Radiology, University Clinic of Tübingen, Tübingen, Germany; 20 Department of Pediatric Radiology, Hannover Medical School: Medizinische Hochschule Hannover, Hannover, Germany; 21 Department of Radiology, Institute of Diagnostic and Interventional Radiology and Neuroradiology, University Clinic of Essen, Essen, Germany; 22 Division of Hepatobiliopancreatic Surgery, Liver and Kidney Transplantation, Bambino Gesù Children’s Hospital, Rome, Italy; 23 Department of Pediatric Radiology, Geneva University Hospitals: Hopitaux Universitaires Geneve, Geneva, Switzerland; 24 Department of Radiology, Hôpital Femme Mère Enfant - Hospices Civils de Lyon, Bron, France; 25 Department of Pediatric Radiology, Leeds Children’s Hospital, Leeds, UK; 26 Department of Visceral Transplant Surgery, Universitätsklinikum Hamburg-Eppendorf, Hamburg, Germany; 27 Department of Pediatric Gastroenterology and Hepatology, Universitätsklinikum Hamburg- Eppendorf, Hamburg, Germany; 28 Department of Pediatric Radiology, Aix Marseille University, Hôpital Timone Enfants, Marseille, France

**Keywords:** Child, Computed tomography, Liver transplantation, Magnetic resonance imaging, Ultrasonography

## Abstract

**Background:**

Liver transplantation is the state-of-the-art curative treatment for end-stage liver disease. Imaging is a key element in the detection of postoperative complications. So far, limited data is available regarding the best radiologic approach to monitor children after liver transplantation.

**Objective:**

To harmonize the imaging of pediatric liver transplantation, the European Society of Pediatric Radiology Abdominal Taskforce initiated a survey addressing the current status of imaging including the pre-, intra-, and postoperative phases. This paper reports the responses related to postoperative imaging.

**Materials and methods:**

An online survey, initiated in 2021, asked European centers performing pediatric liver transplantation 48 questions about their imaging approach. In total, 26 centers were contacted, and 22 institutions from 11 countries returned the survey.

**Results:**

All sites commence ultrasound (US) monitoring within 24 h after liver transplantation. Monitoring frequency varies across sites, ranging from every 8 h to 72 h in early, and from daily to sporadic use in late postoperative phases. Predefined US protocols are used by 73% of sites. This commonly includes gray scale, color Doppler, and quantitative flow assessment. Alternative flow imaging techniques, contrast-enhanced US, and elastography are applied at 31.8%, 18.2%, and 63.6% of sites, respectively. Computed tomography is performed at 86.4% of sites when clarification is needed. Magnetic resonance imaging is used for selected cases at 36.4% of sites, mainly for assessment of biliary abnormalities or when blood tests are abnormal.

**Conclusion:**

Diagnostic imaging is extensively used for postoperative surveillance of children after liver transplantation. While US is generally prioritized, substantial differences were noted in US protocol, timing, and monitoring frequency. The study highlights potential areas for future optimization and standardization of imaging, essential for conducting multicenter studies.

**Supplementary Information:**

Supplementary material is available at 10.1007/s00247-023-05842-z.

## Introduction

Liver transplantation is the state-of-the-art curative therapy for end-stage liver disease in children. Advances in organ procurement, surgical techniques, and immunosuppression have led to excellent short- and long-term results with a 5-year patient survival rate exceeding 85% [1–3]. Imaging methods are key elements for transplantation programs as they have been shown to assist surgical planning, to guide intraoperative surgical technique, and can be effectively applied to detect post-operative complications [4–10].

During the postoperative period after liver transplantation, the main role for medical imaging is to assist in detecting and managing complications. Biliary and vascular problems are relatively frequent and are more frequently encountered in children than in adults due to smaller anatomical structures, caliber mismatch between donor and recipient vasculature, and the higher rate of split organs used [11–13]. Ultrasound (US) is considered the main modality in post-liver transplant imaging and can be used at the bedside to monitor children after liver transplantation [14]. However, large differences are noted in how exactly the basic US regimen is implemented at the transplantation sites, and when more invasive cross-sectional imaging modalities like computed tomography (CT) or magnetic resonance imaging (MRI) are utilized [15–21].

So far, only limited data exists regarding the optimal setup for postoperative monitoring of children after liver transplantation, and how it should interlink with the preoperative and intraoperative phases. In an attempt to harmonize perioperative imaging among the European centers for pediatric liver transplantation, the European Society of Pediatric Radiology (ESPR) Abdominal Taskforce initiated an online survey addressing the practices of pre- [22], intra- [23], and postoperative imaging. This paper reports the responses on the postoperative imaging section of the survey in order to find a common basis for later consensus recommendations as well as for multicenter studies.

## Material and methods

### The survey

For this online survey, the ESPR Abdominal Taskforce contacted European centers for pediatric liver transplantation asking about their current protocols regarding diagnostic imaging procedures. The survey followed a multidisciplinary approach, and the questions were directed towards all pediatric disciplines involved (e.g., radiology, transplantation surgery, gastroenterology, intensive care). A representative for each center was asked to gather the information from all sub-disciplines and to fill out the online survey using Google Forms. The survey was initiated in 2021 and participating centers were asked to specify their liver transplantation numbers and choice of modalities for the period 2018–2020. A total of 48 questions were organized in six sections: demographics (seven questions), pre-transplant evaluation (eight questions), intraoperative imaging (eight questions), postoperative imaging (15 questions), liver elastography (six questions), and outlook (four questions). The survey questions including the questions on postoperative imaging, liver elastography, and outlook can be found in Supplementary Material 1. Further information on the participating centers and European site demographics can be found in the paper reporting the responses related to preoperative imaging [22].

## Results

### Ultrasound monitoring

#### Departmental responsibility and level of expertise

**Fig. 1:**
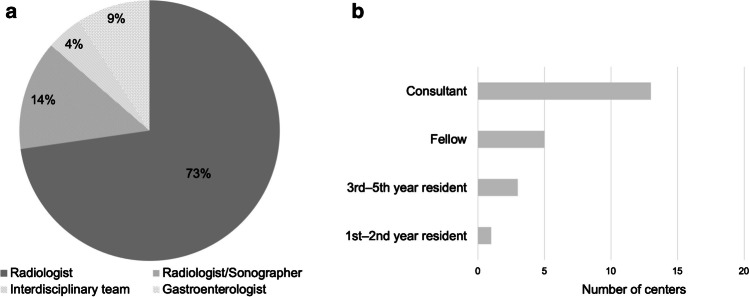
Disciplines (**a**) and operator expertise (**b**) for postoperative ultrasound monitoring in children after liver transplantation*.* Answers from 22 European centers. Level of expertise is according to general level of training in the medical discipline

Radiologists are responsible for the postoperative US examination at most sites (19 of 22 sites, 86.4%), while at one site, postoperative US is carried out as part of an interdisciplinary team. At the remaining two hospitals (9.1%), US examinations were performed by gastroenterologists. The level of medical training of the person performing the bedside US is generally high (Fig. 1). At four sites, the US is primarily performed by radiology residents. These are supervised by consultants which is an indication of the specialized training involved.

#### Availability of intraoperative information

Before conducting the first US, information regarding the intraoperative course and the specific transplant anatomy are obtained from the surgical report at most sites (18 of 21, 85.7 %). A drawing on paper showing the anastomosis is also produced at nine sites (42.9%). The intraoperatively stored US examinations are less frequently used as a source for postoperative information or as a baseline for comparison (4/21 sites, 19.0%).

#### Timing

**Fig. 2:**
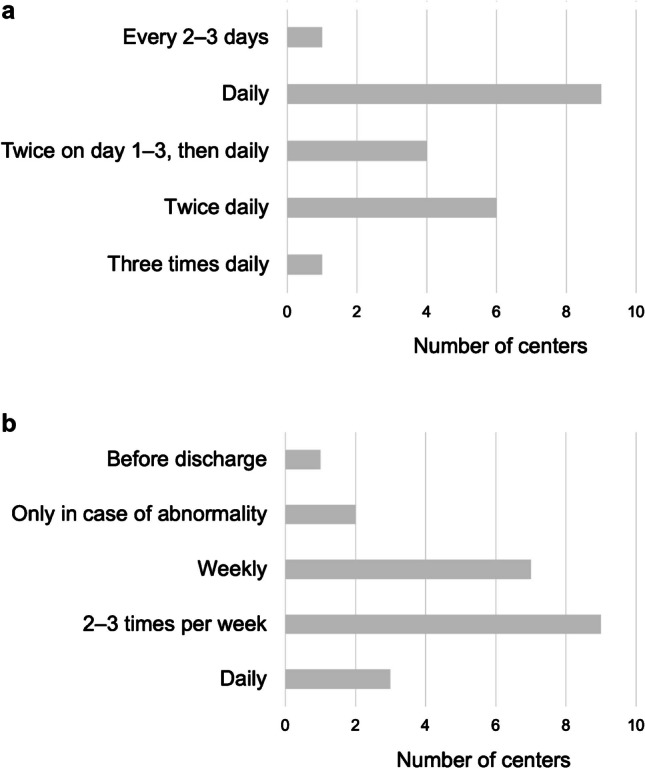
Ultrasound monitoring frequency during the early (days 1–7) postoperative (**a**) and late (>day 7) postoperative (**b**) phases, **a** Answers from 21 European centers. **b** Answers from 22 European centers

The first postoperative US scan is performed within 24 h after the liver transplantation at all sites. Most sites perform this first US examination either directly upon arrival on the intensive care unit (ICU, 8/21 sites, 38.1%) or within the first 6–12 h after the operation (9/21 sites, 42.9%). Subsequently, during the early postoperative phase (within the first 7 days), US assessments are scheduled at relatively short intervals, ranging from every 8 h up to every 48–72 h (Fig. 2). During the late postoperative phase (>7 days after liver transplantation), scheduled intervals are widened ranging from daily US scans to a single scan before discharge (at one site). A minority of sites do not regularly screen with US during the late postoperative phase but image only in case of clinical abnormalities or when follow-up has been requested because of previous abnormalities on imaging (2/22 sites, 9.1%, Fig. 2).

### Protocol elements

**Fig. 3:**
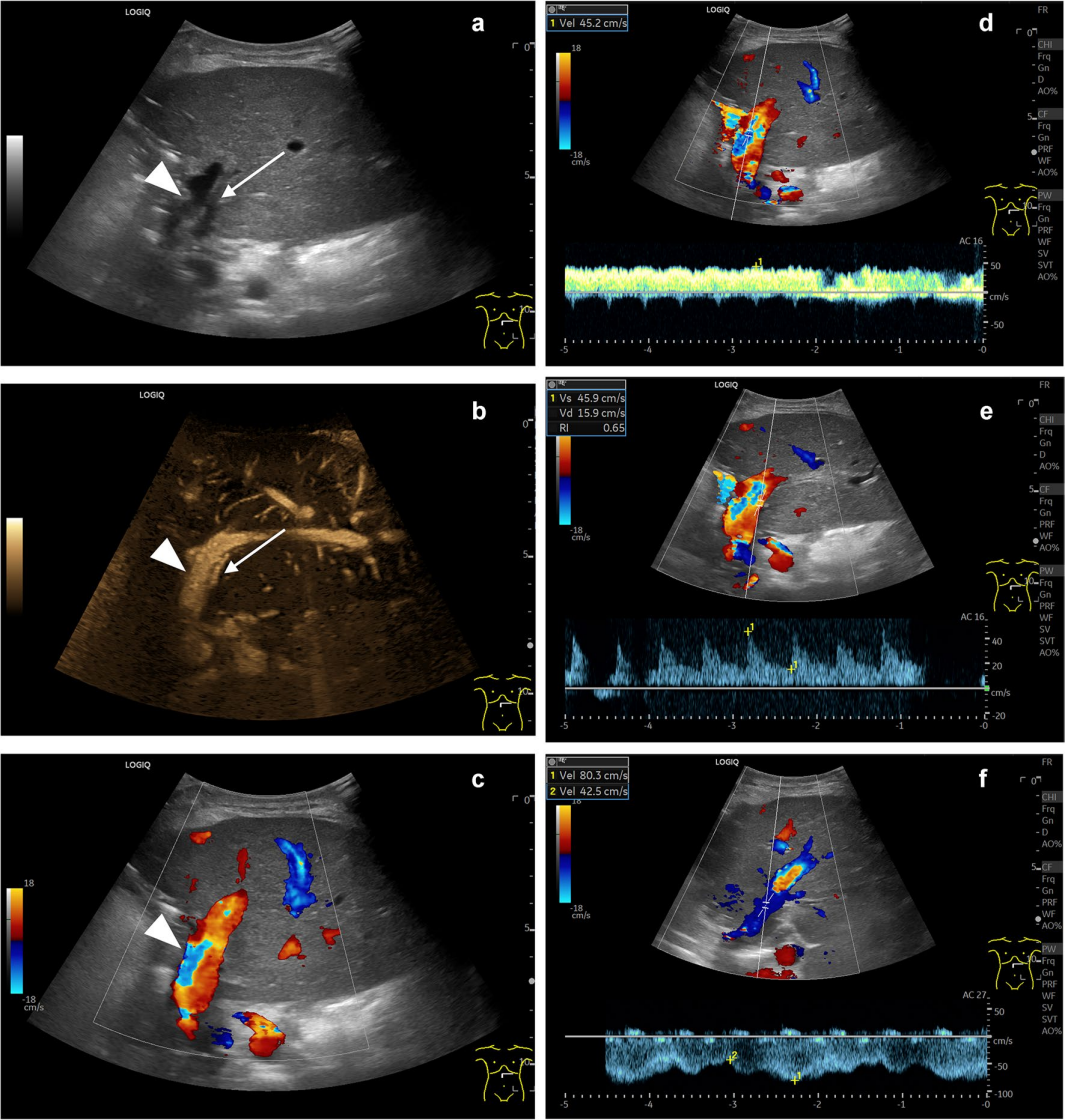
Postoperative ultrasound in transverse plane on day 21 after combined kidney and liver transplantation in an 8-year-old girl with a diagnosis of methylmalonic acidemia. The left-lateral split liver transplant (segments 2, 3) is shown in the upper abdomen. On B-mode (**a**) and B-flow (**b**), the neighboring portal vein (*arrowheads *in **a** and **b**) and hepatic artery (*arrows *in **a** and **b**) can be seen entering the neo-hilum. On the color Doppler image (**c**), the hepatic artery is not distinguishable from the portal vein as the color signal extends beyond the vessel’s true boundaries (blooming artifact). Regular flow patterns are noted in the portal vein (**d**), hepatic artery (**e**), and hepatic vein (**f**)

**Fig. 4:**
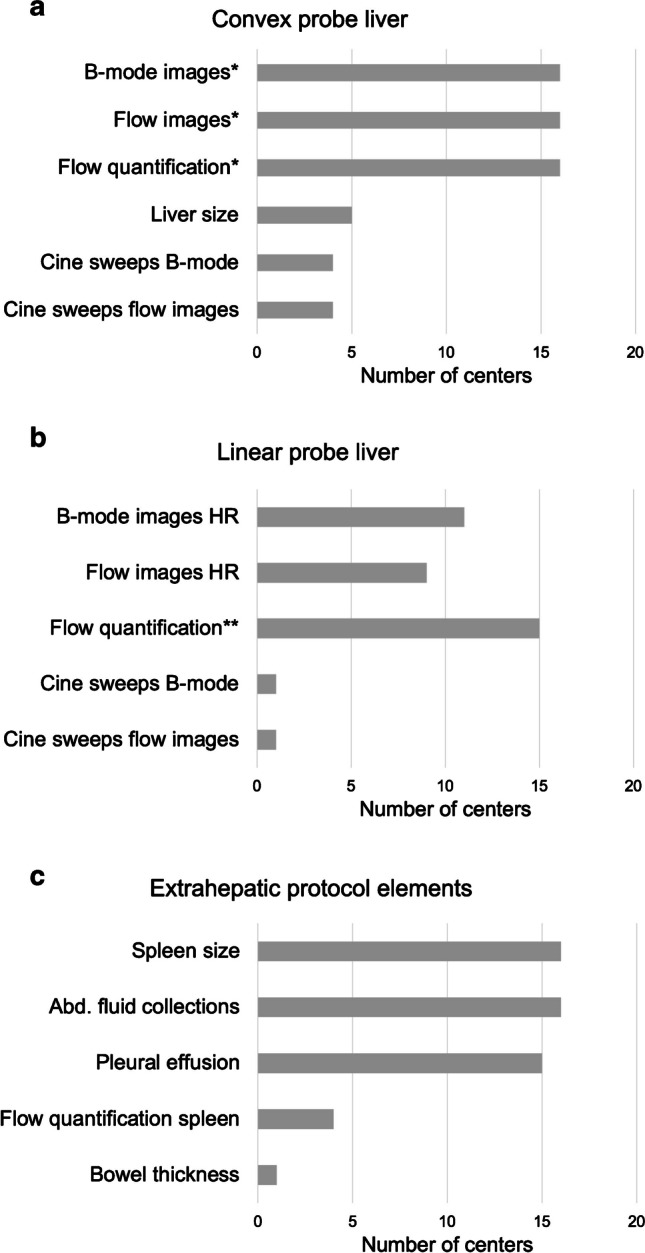
Protocol elements used for postoperative ultrasound monitoring after pediatric liver transplantation (answers from 16 centers with defined protocols). **a**, **b** Hepatic protocol elements using a convex (**a**) and linear probe (**b**). **c** Extrahepatic protocol elements. *At neo-hilum and outflow tract, **at intraparenchymal level; *abd.* abdominal, *HR* high resolution

A predefined US protocol to examine children after the liver transplantation is used by 16 of 22 sites (73%). All protocols uniformly incorporate single gray scale and color Doppler images of the neo-hilum and the outflow tract, along with quantitative flow measurements of the hepatic artery, portal vein, and hepatic veins (Figs. 3, 4). In addition, some sites store US volumes (cine sweeps) covering the main parts of the organ or specific areas and produce high-resolution images using linear probes (Fig. 4).

**Fig. 5:**
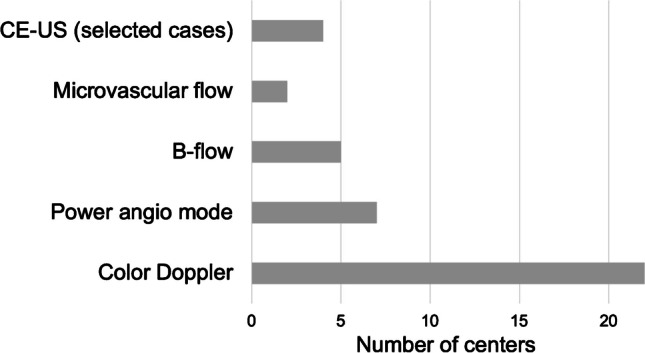
Ultrasound techniques used for visualization of vessels. *CE-US* contrast-enhanced ultrasound

The main flow imaging technique is color Doppler US. Alternative non-Doppler-based applications as well as contrast-enhanced US (CE-US) are used by relatively few sites (Fig. 5). CE-US is used by four of 22 sites (18.2%) during the postoperative phase in doubtful cases when vascular complications such as thrombosis of the hepatic artery or portal vein are suspected. A non-imaging-based device is occasionally used only at one center (4.5%) in the form of a microdialysis catheter placed within the transplant, measuring metabolic substances to detect ischemia and rejection during the early postoperative phase [24].

#### Liver stiffness measurement

US elastography is available at most of the transplant centers for the assessment of liver stiffness during the post-transplant period (14/22 sites, 63.6 %). This involves 2-dimensional (D) shear wave elastography (10/14, 71.4 %), point shear wave elastography (3/14, 21.4%), and transient elastography (3/14, 21.4%). Two sites use point shear wave and 2-D shear wave elastography.

Most centers have no rule as to when they schedule the first stiffness measurement or how often it is repeated during follow-up. Two centers consider US elastography an integral element of their routine post-transplant imaging protocol and use it approximately every 24 h during the first 7 days.

The majority of centers using US elastography do not require children to have fasted before liver stiffness measurement during the postoperative phase (8/14 centers, 57.1%). Four centers require a 2-h fasting period for all children (3/14 centers, 21.4%) and one center a 4-h fasting period (1/14%, 7.1%). Two centers demand fasting before US elastography only in non-intensive care and outpatient settings (2/14 sites, 14.3%).

MRI elastography is available only at one European center (1/22, 4.5%).

#### Computed tomography

**Fig. 6:**
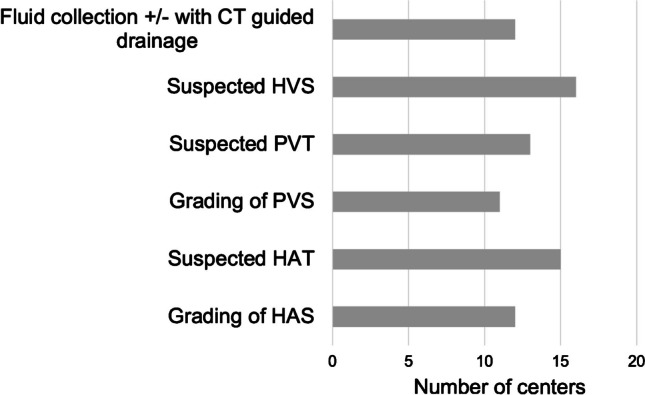
Indications for postoperative computed tomography (CT). *HAS*, hepatic artery stenosis; *HAT*, hepatic artery thrombosis; *HVS*, hepatic vein stenosis; *PVS*, portal vein stenosis; *PVT*, portal vein thrombosis

Postoperative abdominal CT is performed at 19 of 22 (86.4%) centers after liver transplantation when further clarification is needed. The main indications are suspected vascular problems of hepatic inflow and outflow on postoperative US scans, as well as intra-abdominal fluid collections needing CT-guided drainage (Fig. 6). During the study period, a total of 109 abdominal CT examinations per year were performed in children after liver transplantation. This means that approximately 21.5% of children required a CT following transplantation (109/508). The proportion of children who required a postoperative CT differed substantially between the European centers (median postoperative CT rate per liver transplantation, 0.18; range, 0–1.86).

#### Magnetic resonance imaging

**Fig. 7:**
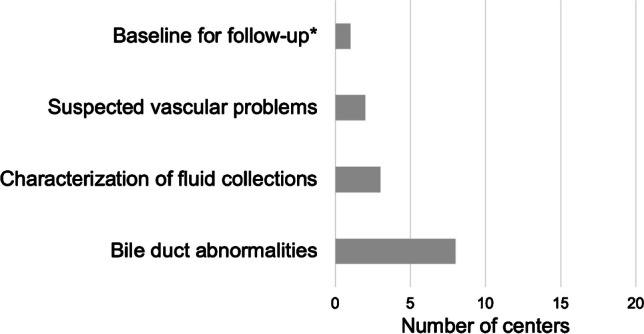
Indication for postoperative magnetic resonance imaging. *In non-sedated children

Postoperative abdominal MRI is used in selected cases in about one-third of the sites (8/22 sites, 36.4%). The main indication to perform MRI is a suspected bile duct abnormality on US with the need for further characterization with magnetic resonance cholangiopancreatography (MRCP) in 8/8 (100%) sites using MRI. Intra-abdominal fluid collections and vascular problems are additionally examined with MRI at three and two sites, respectively (3/8 sites, 37.5%; 2/8 sites, 25%). Only one site performs MRI as part of the routine post-transplant imaging at the end of the postoperative phase in children that do not require sedation (1/22 sites, 4.5%) (Fig. 7).

## Discussion

This paper presents the postoperative results of a multicenter European survey investigating the current practices regarding pediatric liver transplantation imaging. The pre- and intraoperative findings are reported separately [22, 23]. This survey among 22 European sites for pediatric liver transplantation demonstrates that diagnostic imaging is an integral part of the postoperative surveillance after transplantation. US including color Doppler US is used to screen children at bedside in all sites, whereas CT and MRI are only sporadically used as second-line problem solving modalities. This stepwise diagnostic approach is well supported by the literature showing that monitoring with imaging is important to detect postoperative complications which will guide management to help maintain transplant viability [8, 14]. Apart from the conceptual similarities, the survey also identified large differences in the way postoperative monitoring is implemented at the sites.

At all transplant centers, US monitoring is started within 24 h after the liver transplantation and carried out with relatively high frequency during the first week, reflecting the fact that most vascular complications usually occur early after the operation [25]. However, we noted a large discrepancy in how soon the first US on the ICU is scheduled, and how intensively further monitoring is pursued. During the early postoperative phase, the US imaging frequency ranged from three times daily to once every 3 days, and in the later postoperative phase (>7 days after liver transplantation) from daily controls to only sporadic examinations. Similarly, different monitoring protocols can be deduced from published international studies [20, 26–34].

One of the possible reasons for variable monitoring intensities between the sites are differences in local risk factors for some of the postoperative complications. From the survey, we know that the variables of age, underlying diseases, and types of transplanted organs were unevenly distributed, indicating a high level of specialization. It is known that younger patients (newborns/infants), high preoperative Pediatric End-stage Liver Disease (PELD) scores, deceased donor grafts, fulminant liver failure, and liver diseases in the absence of underlying chronic liver disease (e.g., metabolic disorders, hepatoblastoma) constitute an increased risk for postoperative complications and adverse outcomes requiring special attention [35–42].

However, it is also crucial to acknowledge that high-frequency US monitoring regimes which promise a higher level of diagnostic certainty are also relatively labor-intensive. The centers predominantly allocate their experienced personnel to these bedside controls which can take about 20 min to 30 min for a single examination. Maintaining image quality, continuity, and a clear role for US within a diagnostic algorithm seems important, and most institutions lay the responsibility for postoperative bedside US monitoring on their radiology departments, which then need to be staffed accordingly.

Defining standards for image acquisition, documentation, and reporting of the bedside US creates the precondition for an interdisciplinary approach and improved multicenter cooperation. This survey showed that currently there is no full agreement on how to perform the postoperative US examination, and effectively only 73% of the sites reported the use of a predefined image acquisition protocol. The most used protocol elements consist of representative gray scale and color Doppler flow images, along with the measurement of flow velocities at the neo-hilum and of the outflow tract using a convex transducer, as well as of certain extrahepatic elements.

Some sites regularly or optionally use a broader documentation by recording US volumes of the transplant, applying high-resolution linear transducers, using different vascular imaging techniques, and including graft or spleen elastography. Alternative non-Doppler-based imaging techniques offer advantages for vessel delineation attributable to a higher temporal and spatial resolution, a better dynamic range, reduced angle dependency (Fig. 3), and low flow detectability [43–46]. Liver and spleen stiffness can be substantially increased after liver transplantation as in cases with hepatic outflow obstruction, rejection, and infection [47–51]. But so far, there is little systematic data available in children regarding the postoperative baseline liver stiffness values and cutoffs for pathologic changes using the various elastography methods [52]. A multiparametric approach applying the full bandwidth of US techniques for extended hepatic evaluation is not yet consensus among the European sites in the post-transplant monitoring scenario [53].

Additional cross-sectional imaging is used as a problem-solving technique when specific complications occur or are suspected. Abdominal CT is effectively used in about one-fifth (21.5 %) of all pediatric liver transplantations during the postoperative phase. Some centers utilize abdominal CT imaging more frequently than others, which may be attributed to variations in the occurrence of certain postoperative complications, availability of CT at short notice, local practices, and the use of alternative diagnostic pathways. The main indications to perform a postoperative abdominal CT were either suspected vascular problems (ruling out thrombosis, grading of stenosis) or characterization of fluid collections not well seen on US, possibly with CT-guided drainage. Especially in smaller children, some centers will directly pursue a repeat operation when there is a high index of suspicion for hepatic artery thrombosis on Doppler US. CE-US, which has been suggested as a bedside alternative to CT to rule out hepatic artery or portal vein thrombosis, is routinely used at only four European sites participating in this survey (18.2%) [54–56]. Postoperative abdominal MRI is applied by two sites to further assess vascular problems (9.1%). The general availability of MRI is lower than CT (eight sites, 36%), and abdominal MRI is predominantly applied for characterization of biliary problems representing the most frequent complications during this period.

This and the other two papers [22, 23] on perioperative imaging in pediatric liver transplantation are subject to several limitations, some of which are inherent to the use of survey data including issues with respondent selection and response accuracy. (1) Of the 26 European centers for pediatric liver transplantation known to the Abdominal Taskforce, 22 returned the survey. The papers only reflect the views of centers who participated in the survey. Taking a survey return rate of 84% into account, the number of pediatric liver transplantation performed at the participating centers aligns with the numbers published from the European Liver Transplant Registry Database and indicates the sample of invited institutions to be representative [2]. (2) Issues with response accuracy and incomplete coverage were addressed by callbacks. (3) A single respondent was asked to fill out the survey questions representing the position of their center and coauthored the manuscripts to ensure that these views are adequately presented. However, the responses may not fully reflect the position of each medical discipline in detail. To arrive at valid imaging recommendations applicable to all medical disciplines, specific needs and constraints have to be addressed through a consensus approach.

In conclusion, the European centers for pediatric liver transplantation attach high importance to imaging methods during the perioperative period, with US being widely accepted as the primary imaging tool. The comprehensive utilization of multiparametric US techniques for longitudinal assessment is not yet standard practice but may enhance both the diagnostic and prognostic capabilities of US in the future, potentially reducing the need for more invasive imaging. The participating centers stated a high motivation to further cooperate in order to homogenize the approach to imaging post liver transplantation. Our next goal will be to work on a consensus including all disciplines defining both the obligatory and optional elements of US monitoring and diagnostic algorithms post-liver transplantation in children.

### Supplementary information

ESM 1 (PDF 148 kb)

## Data Availability

The datasets generated during and analyzed during the current survey research are not publicly available as individual privacy was guaranteed to all participating centers. Blinded data are however available from the authors upon reasonable request and with permission of all participating centers.
